# SiteRx AI‐Enabled Clinical Intelligence Platform Delivers Superior Amyloid PET Conversion in Early Alzheimer’s Disease (AD) Trial

**DOI:** 10.1002/alz.094711

**Published:** 2025-01-09

**Authors:** Erin Beck, Frank Perilli, Michael Stalder

**Affiliations:** ^1^ SiteRx, New York, NY USA

## Abstract

**Background:**

Accurate participant selection is pivotal for the success of Alzheimer’s Disease (AD) clinical trials, particularly in studies requiring confirmation of amyloid plaque presence via PET scans. SiteRx’s AI platform, InclusionAI, enabled 2.7x higher amyloid PET pass rates1 compared to other sources, delivering a significant enrollment contribution to an ongoing Phase 1 study in Early AD (the “Study”).

**Method:**

SiteRx democratizes access to clinical research via the largest neuro focused real world data network in the US. The SiteRx AI‐enabled process leverages structured and unstructured data from longitudinal health records and relevant data against trial inclusion/exclusion criteria to match candidates to trials. The SiteRx platform captures clinical insights and detailed recruitment funnel conversion across screening activities, including amyloid PET. This research compares outcomes between the SiteRx platform and all other study recruitment sources, including site databases and central advertising campaigns.

**Result:**

SiteRx patients demonstrated an amyloid PET pass rate of 64%, significantly exceeding the 23% pass rate from other sources—a testament to the predictive power of SiteRx AI‐enabled clinical intelligence. This effectiveness translated to savings of approximately $16,200 per patient in PET scan costs, amounting to over $3 million for the patients screened to date. Additionally, 85% of SiteRx’s PET‐eligible patients progressed to randomization, reflecting a higher readiness and suitability for trial participation compared to 66% from all other sources. The cumulative benefits of SiteRx have contributed more than a full Study cohort of randomizations.

**Conclusion:**

SiteRx InclusionAI platform underscores a scalable and economically potent approach to AD clinical research recruitment. Access to longitudinal medical records and other relevant data such as previous brain imaging, cognitive inventory exams (e.g. MMSE, MoCA), current medications, documented ICD codes and formal AD diagnoses drive superior amyloid PET pass rates. This data showcases how targeted, data‐driven recruitment strategies can profoundly impact AD recruitment quality, efficiency and speed for clinical trial sponsors.

